# Probiotic-enzyme complex improves laying performance in laying hens fed low-protein diets by modulating gut microbiota and host metabolism

**DOI:** 10.3389/fvets.2026.1834201

**Published:** 2026-05-29

**Authors:** Qiuchen Liu, Yan Liu, Chunqiao Shan, Chaoxin Ma, Juan Li, Guotuo Jiang

**Affiliations:** 1 Dalian Sanyi Biotechnology Research Institute, Dalian Sanyi Animal Medicine Co., Ltd., Dalian, China; 2 College of Animal Science and Veterinary Medicine, Shenyang Agricultural University, Shenyang, China; 3 Research Quality Control Center, Jiangsu Sanyi Animal Nutrition Technology Co., Ltd., Xuzhou, China

**Keywords:** gut microbiota, laying hens, laying performance, low-protein diet, metabolism, probiotic-enzyme complex

## Abstract

This study was conducted to evaluate the impacts of a probiotic-enzyme complex (PEC) on laying performance, egg quality, intestinal health, and serum metabolite levels in laying hens fed a low-protein diet. A total of 720 Hy-Line Brown laying hens aged 45 weeks were randomly allocated into three groups, with six replicates per group and 40 hens per replicate. The experiment lasted for 10 weeks. The high-protein (HP, 17% CP) group was fed a high-protein diet, whereas the low-protein (LP, 15% CP) group received a low-protein diet, and the PEC group was fed the same LP diet supplemented with the 0.1% PEC (consisting of *Bacillus subtilis* K104, *Lactobacillus plantarum* A490, *Clostridium butyricum* DS142, and acid protease). Results indicated that the PEC group exhibited significantly higher egg production rate and average daily egg yield compared to the LP group (*p* < 0.05), while the FCR was significantly lower than that of the LP group (*p* < 0.05). The eggshell strength, jejunal villus height, and villus height-to-crypt depth ratio in the PEC group were significantly higher than those in the LP group (*p* < 0.05). Additionally, the probiotic-enzyme complex enhanced the biosynthesis of arginine and cofactor pathway in laying hens, increasing the relative abundance of beneficial bacteria such as Lactobacillus and Bacillus. In conclusion, supplementing laying hen diets with the probiotic-enzyme complex improves gut health and metabolic function, thereby enhancing laying performance and eggshell quality, and mitigating the adverse effects of low-protein diets in laying hens.

## 1 Introduction

Eggs are a cost-effective and nutritionally dense food source, ranking among the most widely consumed animal products. The egg-laying poultry sector is a vital component of the livestock industry (1). The production efficiency and sustainable development of this sector have a direct impact on both the economic benefits of the industry and food security (2, 3). In recent years, the scarcity of protein feed resources and the intensifying environmental pressure caused by nitrogen emissions during the farming process have led to a growing focus on the use of low-protein diets in livestock and poultry farming (4–6). This approach is regarded as a pivotal strategy for mitigating feed resource conflicts and promoting environmental sustainability.

Protein serves as a key indicator in the realm of dietary nutrition, executing a multitude of physiological functions. It plays an irreplaceable role in maintaining tissue growth and in synthesizing enzymes, hormones, and antibodies. The level of protein in an animal’s diet, the balance of amino acids in the diet, and the digestibility of the protein affect the animal’s performance (7, 8). A reduction in dietary protein levels has been shown to result in decreased production performance in laying hens, manifesting primarily as fluctuations in egg production rates and egg quality. This phenomenon is likely associated with inadequate amino acid supply, nutritional metabolic imbalance, and compromised gut health (6, 9–12). Liu et al. (11) indicated that reducing the dietary crude protein content from 15.63 to 13.85% exerted a significant negative impact on final body weight, average daily gain, egg production rate, and egg weight. Hu et al. (12) reported that, during the late laying period, low-protein diets reduced egg quality, altered cecal microbiota composition, and induced lipid metabolism disorders by upregulating hepatic genes involved in fatty acid synthesis. Consequently, the question of how to sustain or enhance the productivity of laying hens while concurrently reducing dietary protein levels has emerged as a prominent research focus in the domain of animal nutrition.

Regulating the gut microbiota and host metabolic functions through exogenous additives is a key approach to improving animal production performance. Previous studies have shown that probiotics and enzyme preparations exert beneficial effects by promoting animal growth, enhancing gut health, and improving the digestion and absorption of nutrients (13, 14). Probiotics have been demonstrated to modulate the composition of the gut microbiota and improve intestinal barrier function through mechanisms such as competitive exclusion and secretion of metabolic products (15, 16). The utilization of enzyme preparations can address the deficiencies in endogenous digestive enzymes, thereby enhancing the availability of nutrients and reducing feed costs (17, 18). Furthermore, the organic acids produced by probiotics may increase the overall activity of digestive enzymes by activating zymogens such as proteases, thereby achieving a synergistic effect between probiotics and enzymes (19). Probiotic-enzyme complexes, as biological agents combining the synergistic effects of probiotics and enzyme preparations, offer multiple potential advantages in improving animal gut health and enhancing the digestion and absorption of nutrients. Multiple studies indicate that probiotic-enzyme complexes hold potential for improving gut health, enhancing immunity, and promoting nutrient metabolism in broiler chickens (20–22). However, the application effects and mechanisms in laying hens fed low-protein diets remain to be systematically elucidated.

To the best of our knowledge, extant research has chiefly concentrated on the effects of probiotics or enzyme preparations on growth performance and gut microbiota, with limited systematic analysis of their regulatory pathways on laying hen egg production from the perspective of “gut microbiota-host metabolism” interactions. In the context of low-protein diets, the potential of the probiotic-enzyme complex to enhance production performance by modulating the gut microbiota and improving metabolic function requires further elucidation. We hypothesized that adding 0.1% of the PEC to the LP diet would improve gut health and alleviate the decline in production performance caused by inadequate protein intake. To test this, we evaluated the effects of the PEC on the laying performance of hens fed an LP diet. By examining gut microbiota composition and serum metabolomics, we sought to elucidate potential underlying mechanisms, thereby providing theoretical foundations and practical references for developing green and efficient feed additives for laying hens.

## 2 Materials and methods

### 2.1 Experimental design and diets

The protocol for the animal experimental procedures was approved by the Institutional Animal Care and Use Committee of Shenyang Agricultural University (Shenyang, China). The ethics approval number for this study is SYAU-IACUC-2025-0117. A total of 720 healthy 45-week-old Hy-Line Brown laying hens with similar egg production rates were selected and randomly divided into three groups, with six replicates per group and 40 hens per replicate. Laying hens were housed in cages measuring 65 cm in length, 60 cm in width, and 48 cm in front height. Each replicate served as an experimental unit, and each experimental unit consisted of 10 consecutive cage positions. The experimental units of each group were evenly distributed and cross-arranged across the front, middle, and rear areas of the house, as well as between the left and right cage racks and the upper and lower tiers. This arrangement was designed to minimize systematic errors related to light, ventilation, temperature, and cage position, thereby ensuring comparability of environmental conditions among all groups. The control group (HP group, 17% CP) received a HP basal diet, while the first experimental group (LP group, 15% CP) received a LP diet, and the second experimental group (PEC group, 15% CP) received the LP diet supplemented with the 0.1% PEC. The trial lasted for 10 weeks.

The basal diet for the HP group of laying hens was formulated in accordance with NY/T 33-2004. The LP diet (for the LP and PEC groups) was developed according to GB/T 5916-2020, with only the crude protein level lowered while keeping all other nutrient levels consistent with the HP group. The diet formulations were designed to meet or exceed the recommended nutritional requirements for laying hens. The feed composition and nutritional levels are shown in Table 1. The PEC consists of *Bacillus subtilis* K104 ≥ 1.0 × 10^9^ CFU/g, *Lactobacillus plantarum* A490 ≥ 1.0 × 10^9^ CFU/g, *Clostridium butyricum* DS142 ≥ 5.0 × 10^8^ CFU/g, and acid protease ≥ 10,000 μ/g, blended in a 1:1:1:1 ratio on a weight basis. *Bacillus subtilis* and *Clostridium butyricum* were purchased from Jiangsu Sanyi Bioengineering Co., Ltd., while *Lactobacillus plantarum* was obtained from Beijing Haoshiwo Biotechnology Co., Ltd. Acid protease was purchased from Wuhan Xinhua Yang Biotechnology Co., Ltd. The PEC was added to the LP diet as a powder. It was first mixed with the premix and then blended with the other ingredients to improve feed mixing uniformity. Laying hens were managed under consistent conditions with free access to feed and water. Barn temperature was maintained at 22–24 °C, with 16 h of light exposure (10–20 lux).

**Table 1:** Composition and nutrient levels of experimental diets for laying hens (air-dried basis).

Ingredients, %	Groups		Nutrient level^2^		
	HP	LP			
Corn	61.06	68.64	Crude protein, %	17.00	15.00
Soybean oil	1.53	0.25	ME, Kcal/kg	2,750	2,750
Soybean meal	20.94	14.14	Calcium, %	3.80	3.80
Corn protein powder	5.00	5.00	P-total, %	0.57	0.56
CaHPO_4_	1.55	1.59	P-available, %	0.35	0.35
Stone powder	9.18	9.19	Lysine, %	0.78	0.78
NaCl	0.34	0.34	Methionine, %	0.41	0.41
Lysine	0.04	0.33	Threonine, %	0.63	0.63
Methionine	0.10	0.14	Tryptophan, %	0.17	0.17
Tryptophan	0.01	0.04			
Premix^1^	0.25	0.25			
Threonine	0.00	0.09			

1 The premix provides per kilogram of feed: VA 8000 IU, VD3 2,500 IU, VE 25 IU, VK3 2.5 mg, VB1 1.8 mg, VB2 6 mg, VB6 4 mg, biotin 0.06 mg, choline chloride 0.5 g, niacin 35 mg, folic acid 1 mg, pantothenic acid 12 mg, Mn 62 mg, Zn 60 mg, Fe 80 mg, Cu 5 mg, I 1 mg, Se 0.3 mg.

2 Nutrient levels are calculated values.

### 2.2 Sample collection

The laying hens were fasted for 12 h before sampling. Prior to morning feeding, six hens per group (one hen per replicate) were randomly selected, and 2 mL of blood was collected from the brachial vein. Blood samples were allowed to stand for 2 h, and then centrifuged at 3500 rpm for 5 min at 4 °C to obtain serum samples. These were aliquoted into cryogenic tubes, rapidly frozen in liquid nitrogen, and stored at −80 °C for subsequent testing. After blood collection, three laying hens from each replicate were randomly selected and euthanized by cervical dislocation. The duodenum, jejunum, and ileum were rapidly excised. Approximately 2 cm of mid-segment intestinal tissue was excised from each intestinal section and gently rinsed with physiological saline (0.9% NaCl) to remove visible contents, taking care not to damage the mucosa. The rinsed tissues were immediately fixed in a 4% paraformaldehyde solution for 48 h at 4 °C for subsequent histomorphometric analysis (e.g., villus height and crypt depth). The cecum was isolated, and its base was clamped with sterile hemostats. The cecal contents were gently expressed into sterile centrifuge tubes, taking care to avoid contact with the intestinal mucosa to prevent contamination with host DNA. Approximately 1 g per tube was collected, then flash-frozen in liquid nitrogen and stored at −80 °C for microbial sequencing.

### 2.3 Laying performance

During the trial period, under identical conditions for all replicates, data on daily feed consumption, daily egg production, and egg weight were recorded. These data were used to calculate several key performance indicators, including the average daily feed intake (ADFI), egg production rate (EPR), average egg weight (AEW), average daily egg yield (ADEY), and feed conversion ratio (FCR).

### 2.4 Egg quality

At the end of the 10th week of the trial, 10 eggs were randomly collected from each replicate per group (60 eggs per group) for the determination of eggshell color, shape index, strength, thickness, Haugh unit, albumen height, and yolk percentage. The major and minor axes of the egg were measured using a vernier caliper (Deli Group Co., Ltd., DL-9115, China). The ratio of the minor axis to the major axis was the egg shape index. Eggshell color was then measured using a colorimeter (Guangzhou Sanenshi Technology Co., Ltd., YS3060, China). Eggshell strength was measured using an eggshell strength tester (Shanghai Baosheng Industrial Development Co., Ltd., TA.XTC-18, China). The egg was placed on the tester’s support platform, and pressure was applied until the shell was breached. The pressure value at fracture was recorded as the eggshell strength. Eggshell thickness was measured using an eggshell thickness gage (Mitutoyo, 547-400S, Japan). Measurements were taken at three locations on the eggshell: the blunt end, the pointed end, and the equatorial region, and the mean value was then calculated. The Haugh unit and albumen height were determined using an egg quality tester (Shandong Yuntang Intelligent Technology Co., Ltd., YT-DP1, China). The egg was cracked, and the yolk was separated from the albumen using a strainer. The membrane and mucus were removed from the yolk. The yolk was weighed using an electronic balance (Sartorius, Secura 225D-1S, Germany). The yolk percentage was calculated by dividing the yolk weight by the total egg weight.

### 2.5 Intestinal development

The duodenum, jejunum, and ileum were embedded in paraffin blocks and stained with hematoxylin and eosin (H&E). The sections were scanned using a panoramic slide scanner (3DHISTECH, 250 series, Hungary). Once imaging was completed, villus height (VH) and the corresponding crypt depth (CD) were measured at five random locations per section using Image-Pro Plus 6.0 analysis software, in micrometers. The villus height-to-crypt depth ratio (V/C) was then calculated.

### 2.6 Serum metabolomics analysis

Serum samples were retrieved from a −80 °C freezer, thawed, and vortexed for 10 s. An aliquot of 100 μL of serum was precisely transferred into a 1.5 mL centrifuge tube, followed by the addition of 400 μL of extraction solvent (methanol:acetonitrile = 1:1, v/v) spiked with four internal standards (including L-2-chlorophenylalanine at 0.02 mg/mL). The mixture was vortexed for 30 s and then subjected to ultrasonic extraction at 5 °C and 40 kHz for 30 min. The samples were left to stand at −20 °C for 30 min, followed by centrifugation at 13000g and 4 °C for 15 min. The resulting supernatants were collected. After drying under a nitrogen stream, the residues were reconstituted in 120 μL of reconstitution solution (acetonitrile:water = 1:1, v/v), vortexed for 30 s, and ultrasonicated at 5 °C and 40 kHz for 5 min. The reconstituted solutions were centrifuged again at 13000g and 4 °C for 10 min, and the supernatants were transferred into autosampler vials equipped with inserts for subsequent analysis.

LC–MS-based metabolomics analysis was performed by Shanghai Meiji Biomedical Technology Co., Ltd. (Shanghai, China) using a Thermo Scientific UHPLC-Exploris 240 ultra-high performance liquid chromatography-Fourier transform mass spectrometry system. Chromatographic separation was conducted on an ACQUITY UPLC HSS T3 column (100 mm × 2.1 mm i.d., 1.8 μm; Waters, Milford, United States). Mobile phase A consisted of 95% water and 5% acetonitrile containing 0.1% formic acid, while mobile phase B was composed of 47.5% acetonitrile, 47.5% isopropanol, and 5% water with 0.1% formic acid. The injection volume was 3 μL, and the column temperature was maintained at 40 °C. Quality control (QC) samples were interspersed in each batch of serum samples to evaluate system stability and analytical reproducibility.

Raw mass spectrometry data were processed using Progenesis QI v3.0 software (Waters Corporation, Milford, United States). The preprocessing steps included baseline correction, peak detection, integration, retention time alignment, and peak matching to generate a data matrix comprising retention time, mass-to-charge ratio, and peak intensity. Metabolites were identified by comparing MS1 and MS2 spectra against the HMDB and KEGG databases, with a mass tolerance of <10 ppm for MS1 and matching scores for MS2. Missing values were imputed using the minimum value, and data were normalized by total peak area. The preprocessed data were utilized for subsequent differential analysis. Prior to OPLS-DA analysis, data were subjected to log2 transformation and mean centering. To prevent overfitting, 200 permutation tests were performed. Differentially expressed metabolites were screened based on the criteria of FC ≥ 2 or ≤0.5, *p* < 0.05, and VIP ≥ 1, followed by KEGG enrichment analysis.

### 2.7 Microbial analysis of the cecum

Genomic DNA was extracted from the cecal contents of laying hens using the E.Z.N.A.™ Mag-Bind Soil DNA Kit (Omega Bio-Tek, M5635-02, United States). The V3-V4 hypervariable regions of the 16S rRNA gene were amplified via PCR with the universal primers 341F (5′-CCTACGGGNGGCWGCAG-3′) and 805R (5′-GACTACHVGGGTATCTAATCC-3′). A two-round PCR strategy was employed. The first round was carried out using the universal primers in a 30 μL reaction mixture containing 15 μL of high-fidelity PCR Master Mix (American Yisheng Biotechnology (Shanghai) Co., Ltd., 10105ES03, China), 1 μL of each primer, and 20 ng of DNA template, with the volume adjusted to 30 μL using ddH₂O. Thermal cycling conditions were: initial denaturation at 94 °C for 3 min; 20 cycles of denaturation at 94 °C for 20 s, annealing at 55 °C for 20 s, and extension at 72 °C for 30 s; followed by a final extension at 72 °C for 10 min, and held at 4 °C. The second round used Illumina bridge-PCR compatible primers with the same reaction composition, except that the template consisted of the purified PCR product from the first round, and the number of cycles was reduced to five. PCR products were checked for fragment size on a 2% agarose gel, and library concentrations were measured using a Qubit 3.0 Fluorometer (Thermo Fisher Scientific, United States). All samples were pooled in equal amounts (1:1 ratio).

Raw image data generated by Illumina MiSeq™/HiSeq™ sequencing were converted into raw reads through base calling. These raw reads were then merged and quality-filtered using fastp software (v0.20.0) to obtain clean tags. After chimera removal, effective tags were retained for subsequent analysis. Operational taxonomic units (OTUs) were clustered from the effective tags using the UPARSE algorithm (v7.1) at a 97% sequence similarity threshold, and the most abundant sequence in each OTU was selected as the representative sequence. Taxonomic assignment was performed using the RDP Classifier (v2.13) against the SILVA database (release 138) with an 80% confidence threshold. Based on this, microbial diversity, inter-group differences, and relative abundances were analyzed. Non-metric multidimensional scaling (NMDS) was conducted based on the Bray–Curtis distance matrix, and statistical significance was evaluated by analysis of similarities (ANOSIM) with *R* and *p*-values. Relative abundances at the phylum and genus levels were calculated. Differential taxa were identified using linear discriminant analysis effect size (LEfSe, v1.0, LDA threshold = 2) combined with the Wilcoxon rank-sum test (*p* < 0.05), and *p*-values were adjusted using the Benjamini–Hochberg (BH) method. Functional pathways of the microbial community and inter-group differences were predicted using PICRUSt2 (v2.4.1). Additionally, based on genus-level abundance data, a random forest model (R package randomForest, v4.7-1.1) was constructed with ntree = 500 and default mtry to identify key microbial biomarkers. All analytical data were visualized using relevant R packages (v4.2.0).

### 2.8 Statistical analysis

Data were organized using Excel 2021. One-way ANOVA was performed using SPSS 26.0 to compare the overall means (averaged over the 10-week experimental period) among the groups, with each replicate serving as the experimental unit, followed by Tukey’s multiple comparisons test. A repeated measures ANOVA was conducted to analyze the dynamic changes in laying performance over time. Prior to statistical analysis, data were tested for normality and homogeneity of variances using Shapiro–Wilk and Levene’s tests. The EPR data were arcsine-square-root transformed before analysis. Graphs were plotted using GraphPad Prism 8.0.2. All data are presented as mean ± standard error. *p* < 0.05 was considered statistically significant. Metabolomics data were analyzed for differences using an independent samples *t*-test, while microbial data were analyzed using the Kruskal-Wallis test and corrected using the Benjamini-Hochberg (BH) correction. Metabolomics and microbial data visualization were performed using R software (version 4.2.2).

## 3 Results

### 3.1 Laying performance and egg quality

**Figure 1:**
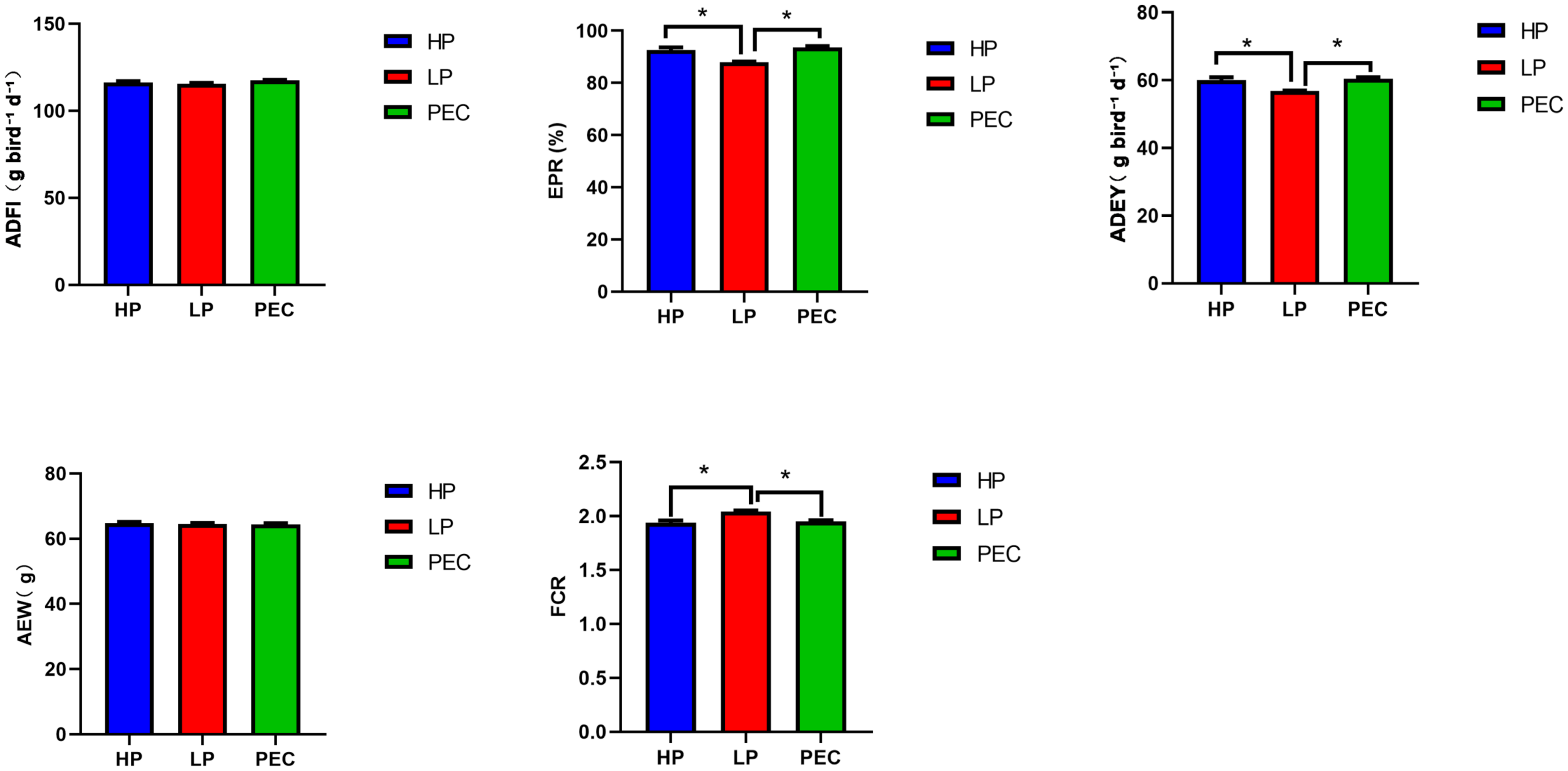
Effect of the probiotic-enzyme complex on laying performance in laying hens throughout the experimental period (*n* = 6). ADFI, average daily feed intake; EPR, egg production rate; ADEY, average daily egg yield; AEW, average egg weight; FCR, feed conversion ratio. LP: 15% protein diet. HP: 17% protein diet. PEC: 15% protein diet + 0.1% probiotic-enzyme complex. *Indicates a significant difference (*p* < 0.05).

**Figure 2:**
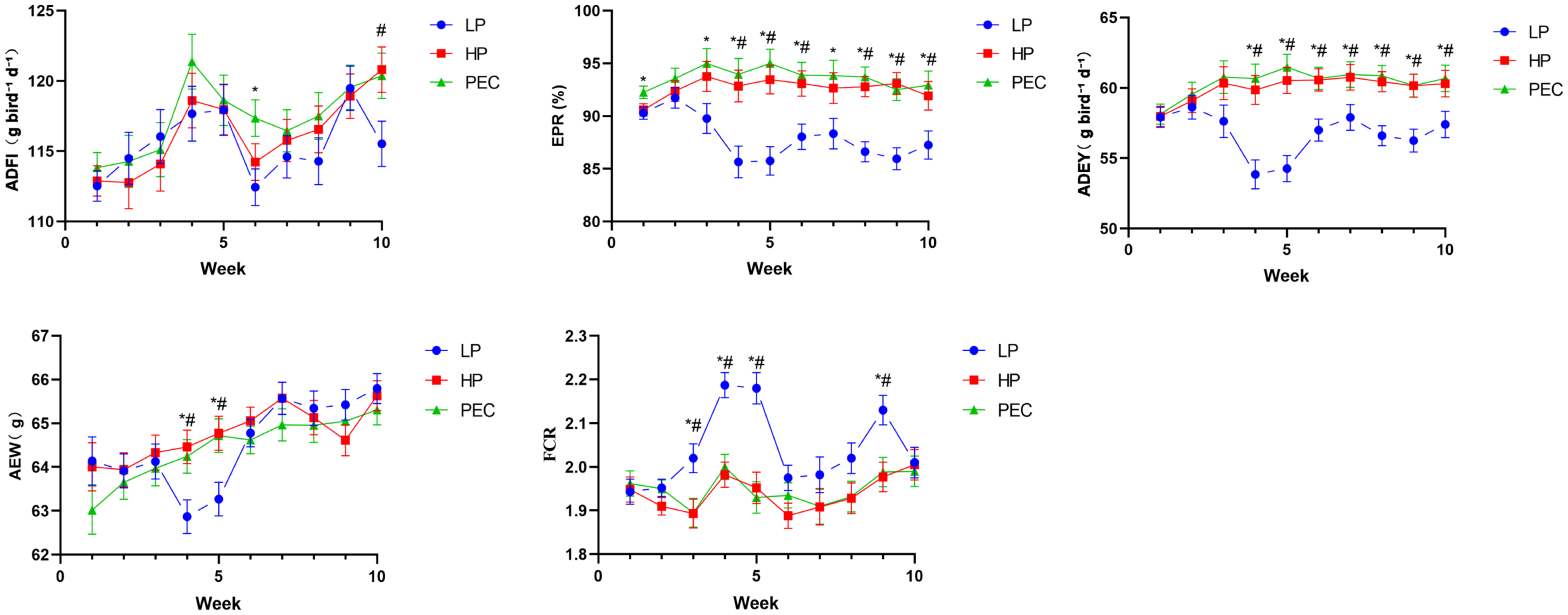
Effect of the probiotic-enzyme complex on weekly laying performance in laying hens (*n* = 6). LP: 15% protein diet. HP: 17% protein diet. PEC: 15% protein diet + 0.1% probiotic-enzyme complex. ^*^Indicates a significant difference between LP and PEC (*p* < 0.05). ^#^Indicates a significant difference between LP and HP (*p* < 0.05).

As demonstrated in Figure 1, supplementation with the probiotic-enzyme complex improved egg production performance and egg quality in laying hens fed a low-protein diet. Specifically, the EPR and ADEY of the HP and PEC groups exhibited significant increases compared to the LP group (*p* < 0.001). Conversely, the F/E of the HP and PEC groups was significantly lower than that in the LP group (*p* < 0.001). Furthermore, as shown in Table 2, the eggshell strength of the PEC group exhibited a significant increase compared to the HP and LP groups (*p* = 0.013). Additionally, the yolk ratio of the HP group was found to be significantly higher than that of the LP group (*p* = 0.032). As shown in Figure 2, except for the second week, the EPR of the PEC group was higher than that of the LP group in all other weeks (*p* < 0.05). Apart from the first 3 weeks, the ADEY of the LP group was significantly lower than that of the HP and PEC groups for each remaining week (*p* < 0.05). The FCR of the LP group was significantly higher than that of the HP and PEC groups in weeks 3, 4, 5, and 7 (*p* < 0.05).

**Table 2:** Effects of dietary supplementation with the probiotic-enzyme complex on egg quality (*n* = 6).

Group	Eggshell color	Egg shape index (%)	Eggshell strength (N/cm^2^)	Eggshell thickness (mm)	Haugh unit	Albumen height (mm)	Yolk percentage (%)
LP	26.7 ± 0.525	78.30 ± 0.338	46.45 ± 0.61^b^	0.37 ± 0.003	76.88 ± 1.552	6.2 ± 0.230	29.91 ± 0.381^b^
HP	25.99 ± 0.498	78.94 ± 0.457	46.61 ± 0.997^b^	0.38 ± 0.004	78.45 ± 1.456	6.39 ± 0.102	31.02 ± 0.257^a^
PEC	26.69 ± 0.789	77.46 ± 0.413	49.7 ± 0.592^a^	0.37 ± 0.003	76.43 ± 0.867	6.18 ± 0.085	30.96 ± 0.237^ab^
*p*-value	0.65	0.062	0.013	0.212	0.539	0.573	0.032

LP: 15% protein diet. HP: 17% protein diet. PEC: 15% protein diet + 0.1% probiotic-enzyme complex. Different letters within the same column indicate significant differences (*p* < 0.05) among means.

### 3.2 Intestinal development

**Figure 3:**
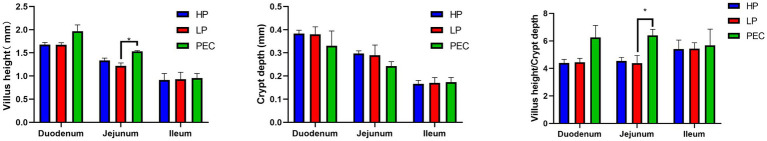
Effects of the probiotic-enzyme complex on intestinal development in laying hens (*n* = 3). LP: 15% protein diet. HP: 17% protein diet. PEC: 15% protein diet + 0.1% probiotic-enzyme complex. ^*^Indicates a significant difference between the two groups (*p* < 0.05).

The effects of the probiotic-enzyme complex on intestinal development are shown in Figure 3. The PEC group exhibited significantly higher jejunal villus height (*p* = 0.010) and villus height-to-crypt depth ratio (*p* = 0.032) compared with the LP group. Intestinal tissue sections are presented in Supplementary Figure 1.

### 3.3 Serum metabolomics

**Figure 4:**
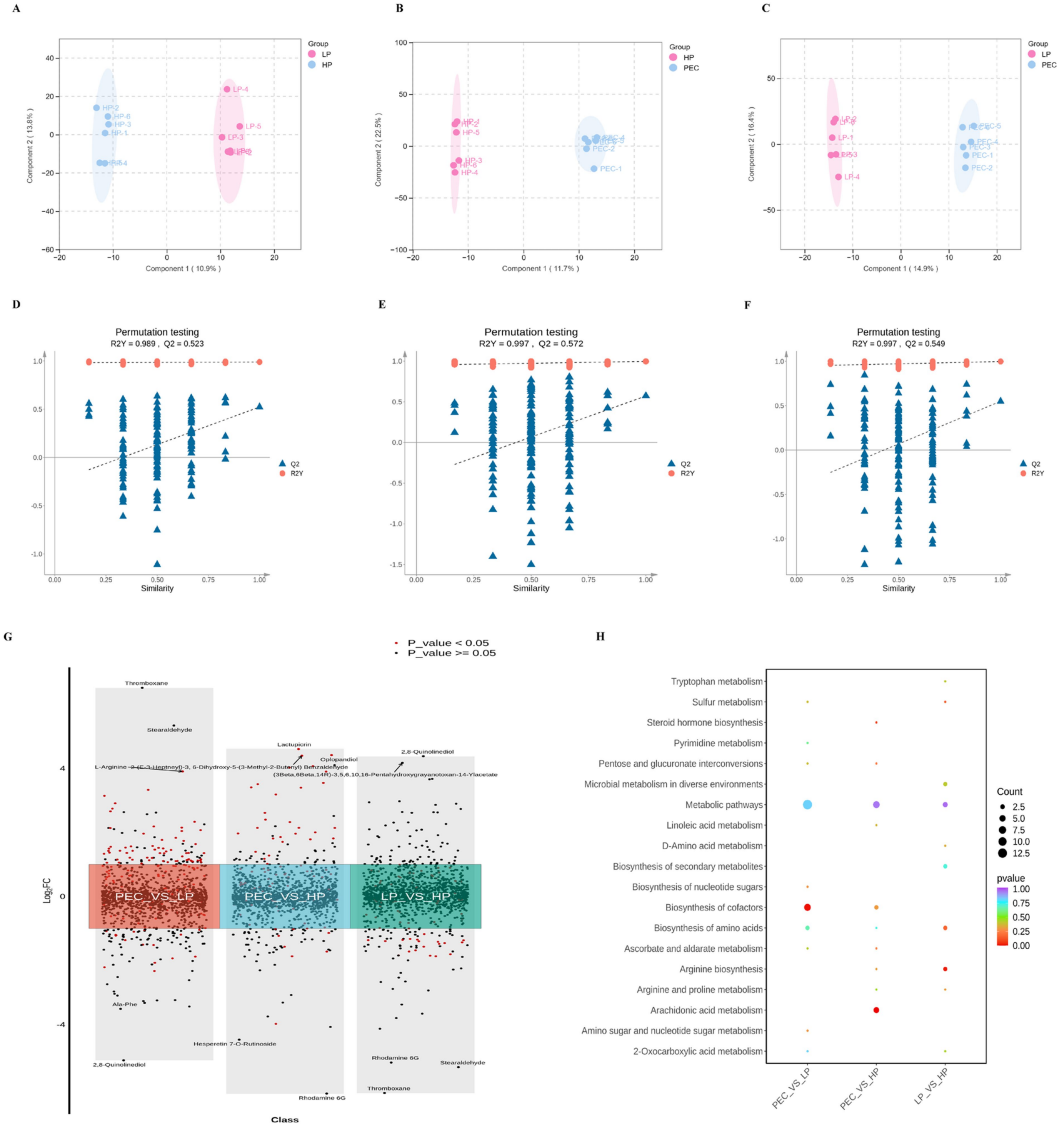
Effects of the probiotic-enzyme complex on serum metabolites in laying hens (*n* = 6). **(A)** OPLS-DA score plot of the HP group vs. the LP group; **(B)** OPLS-DA score plot of the HP group vs. the PEC group; **(C)** OPLS-DA score plot of the LP group vs. the PEC group; **(D–F)** The permutation testing corresponding to **(A–C)**; **(G)** Volcano plot of multi-group differential metabolite combinations; **(H)** KEGG combined enrichment bubble plot.

Figures 4A–C display the OPLS-DA score plots, showing clear separation among the groups. A multi-group differential metabolite combination volcano plot was generated based on FC and *p*-values. Figures 4D–F show the permutation tests corresponding to Figures 4A–C. The permutation test plots indicate that the models for all groups are reliable. As shown in Figure 4G, L-arginine level in the PEC group was significantly increased compared with that in the LP group (*p* < 0.05), while Lactupicrin and Oplopandiol in the PEC group were significantly elevated compared with the HP group (*p* < 0.05). A total of 113 metabolites were identified with the criteria of FC ≥ 2 or ≤ 0.5, *p* < 0.05, and VIP ≥ 1. Specifically, 79 metabolites showed significant differences between the PEC and LP groups, including 63 increased and 16 decreased metabolites (Supplementary Table 1). There were 38 differential metabolites between the PEC and HP groups, including 30 increased and 8 decreased metabolites (Supplementary Table 2). A total of 20 differential metabolites were identified between the LP and HP groups, all of which were decreased significantly (Supplementary Table 3). KEGG enrichment analysis was performed on the differential metabolites subsequently, and the results are shown in Figure 4H. The significantly enriched metabolic pathways included biosynthesis of cofactors, biosynthesis of amino acids, arachidonic acid metabolism, and arginine biosynthesis.

**Figure 5:**
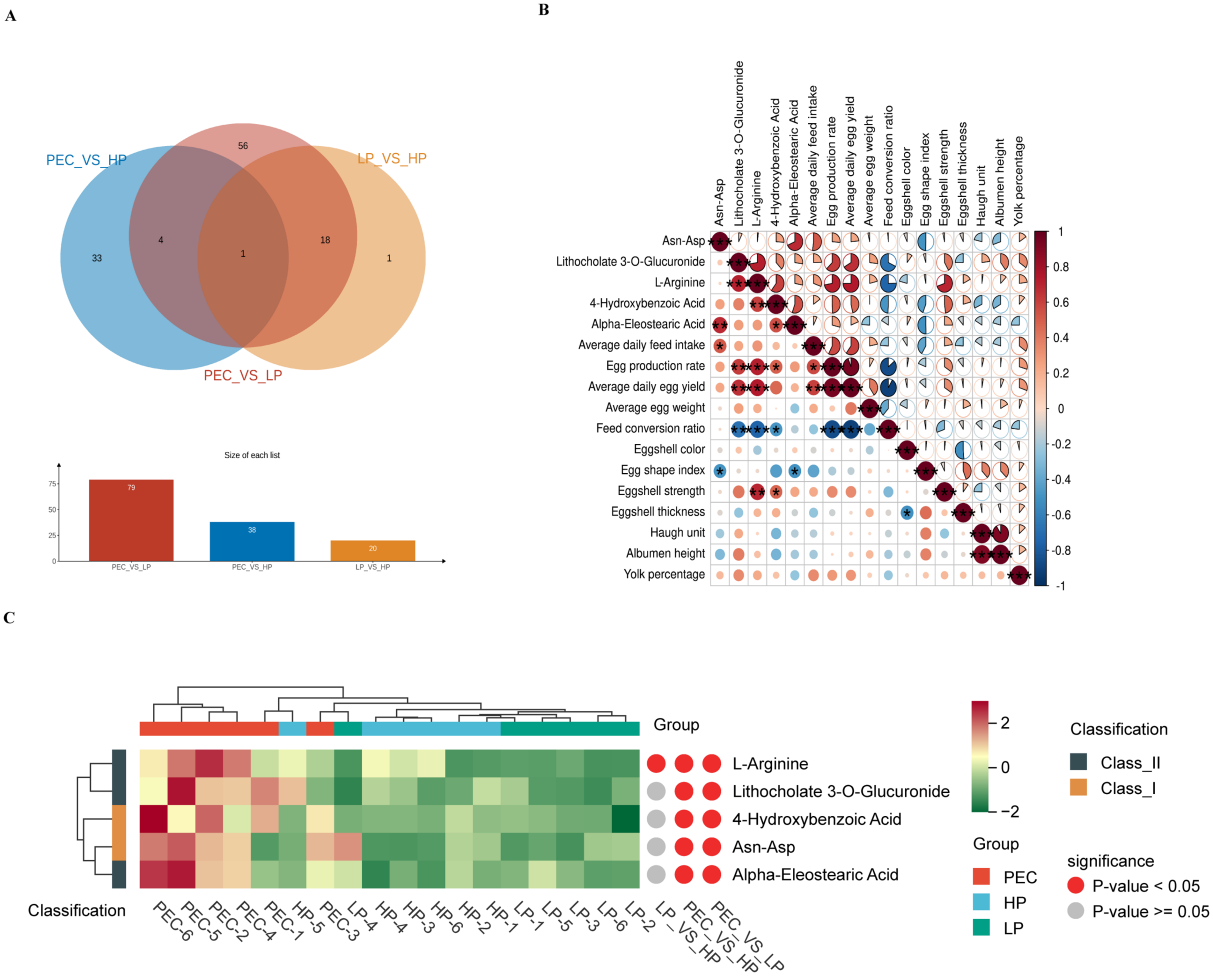
Analysis of significantly differential metabolites (*n* = 6): **(A)** Venn and bar chart of differential metabolites; **(B)** Correlation analysis of common differential metabolites (PEC vs. other groups) with laying performance and egg quality; **(C)** Advanced correlation cluster heatmap of common differential metabolites (PEC vs. other groups). The correlation analysis was performed using Pearson’s method.

The Venn diagram of differential metabolites is shown in Figure 5A. A total of five differential metabolites were identified in the PEC group compared with the other groups, namely Asn-Asp., lithocholate 3-O-glucuronide, L-arginine, 4-hydroxybenzoic acid, and α-eleostearic acid. The correlation analysis between these five differential metabolites and laying performance, as well as egg quality parameters, is presented in Figure 5B. L-Arginine was significantly positively correlated with lithocholate 3-O-glucuronide and 4-hydroxybenzoic acid (*p* < 0.05); L-arginine and lithocholate 3-O-glucuronide were significantly positively correlated with the EPR and ADEY (*p* < 0.05), and significantly negatively correlated with the F/E (*p* < 0.05); eggshell strength was significantly positively correlated with L-arginine and 4-hydroxybenzoic acid (*p* < 0.05). The cluster heatmap of these five differential metabolites in the samples of each group is shown in Figure 5C, with significantly higher abundance observed in the PEC group (*p* < 0.05).

### 3.4 Cecal microbiota

**Figure 6:**
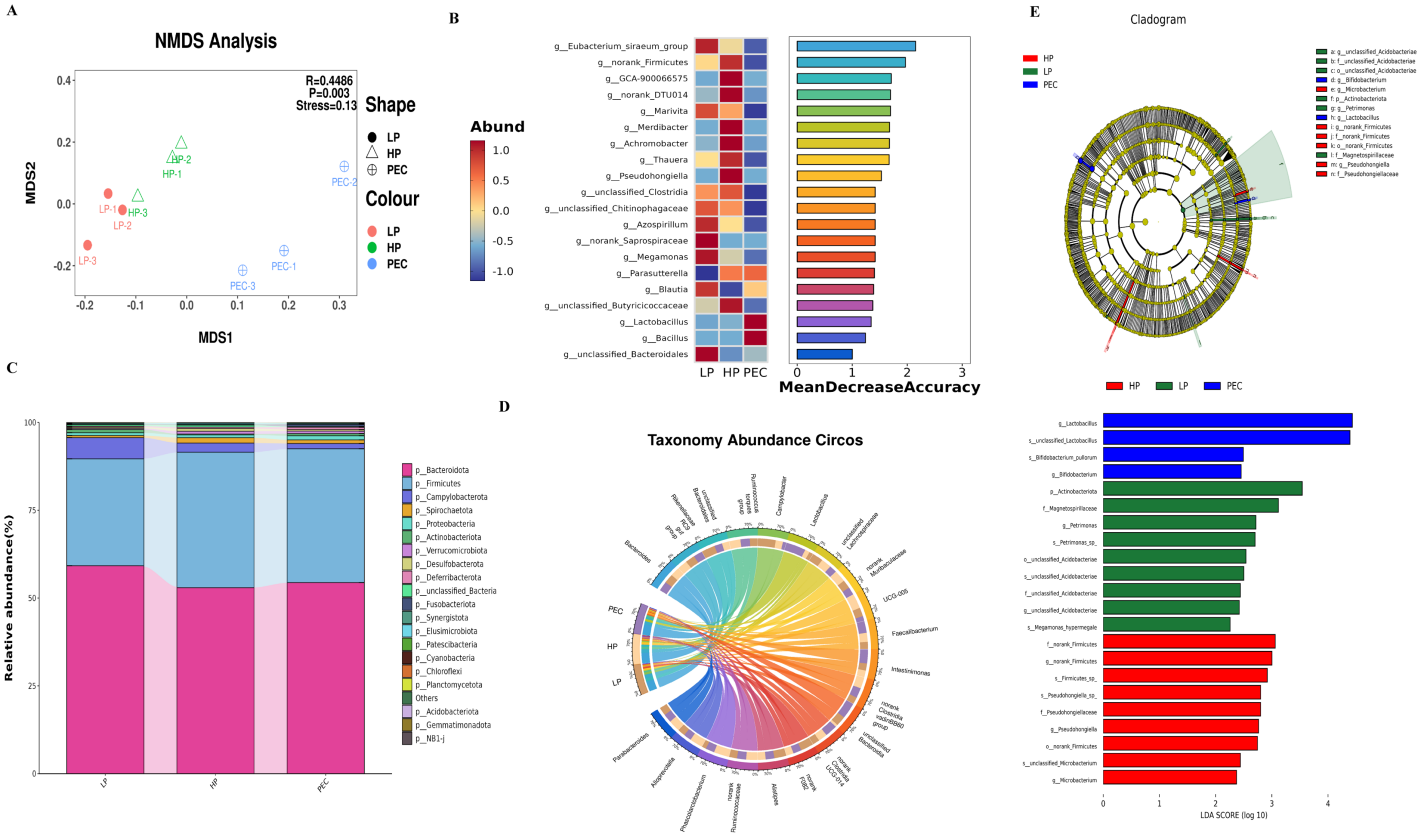
Cecal microbial analysis (*n* = 3). **(A)** Microbial β-diversity analysis; **(B)** Random forest analysis at the genus level; **(C)** Relative abundance analysis at the phylum level; **(D)** Chord diagram at the genus level; **(E)** LEfSe analysis.

The microbial sequencing results indicated no significant difference in the *α*-diversity of the microbial community (Supplementary Table 4). *β*-diversity analysis is shown in Figure 6A, with a stress value of less than 0.2, indicating a good fit, and significant differences were observed among the groups (*p* = 0.003). Random forest analysis at the genus level is presented in Figure 6B, showing enrichment of *g_Lactobacillus* and *g_Bacillus* in the PEC group. The relative abundances at the phylum level are displayed in Figure 6C, with *p_Bacteroidota* and *p_Firmicutes* accounting for more than 80% of the relative abundance in each group. Chord diagram analysis at the genus level is illustrated in Figure 6D, indicating higher relative abundances of *g_Lactobacillus*, *g_Faecalibacterium*, *g_Intestinimonas*, and *g_unclassified Bacteroidia* in the PEC group. The LEfSe analysis results are shown in Figure 6E, demonstrating significant enrichment of *g_Lactobacillus*, *s_unclassified Lactobacillus*, *s_Bifidobacterium_pullorum*, and *g_Bifidobacterium* in the PEC group.

## 4 Discussion

Laying performance and egg quality are key indicators for evaluating layer production. Laying performance directly influences the economic returns of layer farming, while egg quality not only affects resistance to breakage during transport but also determines egg shelf life (23). Probiotics and enzyme preparations, as natural and safe feed additives, have gained increasing attention since the restrictions on antibiotic growth promoters (AGPs) in feed. They contribute to enhancing animal health and improving production performance (17, 23–26). Previous studies have shown that the supplementation of probiotics in layer diets can significantly improve the laying performance, egg quality, and nutrient digestibility of laying hens (27, 28). Yang et al. (29) reported that the supplementation of 0.03% compound probiotics and tea polyphenols in layer diets significantly improved laying performance and egg quality. Wang et al. (23) reported that supplementation with 0.05% compound probiotics significantly enhanced the antioxidant and immune capacities of laying hens, as well as improved their laying performance and egg quality. In addition, Lee et al. (30) found that dietary supplementation with 0.05% enzyme preparations in low-protein and low-energy diets could improve the production performance of laying hens. Huang et al. (31) also confirmed that the supplementation of enzyme preparations in laying hen diets improved the EPR, egg quality, and F/E. A recent study demonstrated that dietary supplementation with probiotics at 1.0 × 10^9^ CFU/kg alleviated the adverse effects of low-protein diets on the growth and development of laying hens to some extent (32). In the present study, the probiotic-enzyme complex improved the EPR, ADEY, and F/E in laying hens fed low-protein diets, which was consistent with the findings of the aforementioned studies. Meanwhile, we also observed an improvement in eggshell strength. In contrast, Khan and Chousalkar (33) reported that supplementation with 0.1% compound probiotics did not improve the EPR, egg weight, and eggshell quality of laying hens, but significantly enhanced the Haugh unit and yolk color. This discrepancy may be attributed to the fact that the efficacy of probiotic application is constrained by multiple factors, including microbial composition, dietary regimens, and the age of laying hens (23).

The improvement of laying hen production performance by the probiotic-enzyme complex may be associated with the enhancement of intestinal health and nutrient digestion and absorption, which are mediated by metabolites produced by probiotics, such as extracellular enzymes, antimicrobial peptides, and short-chain fatty acids (26, 34, 35). In addition, protease preparations may also contribute to enhancing protein digestibility (18, 36–38). Supplementation with exogenous enzyme preparations may enhance digestive enzyme activity and regulate the expression of genes related to intestinal amino acid transporters, thereby increasing crude protein digestibility and exerting a positive effect on laying performance and egg quality in laying hens (31).

The eggshell serves as the primary biological barrier for egg contents; improving eggshell quality and reducing the risk of eggshell breakage are crucial for commercial production (39). Consistent with the results of this study, Wang et al. (40) found that supplementation with 2 × 10^9^ CFU/kg probiotics during the late laying period enhanced intestinal calcium absorption and significantly improved eggshell quality. Likewise, Nishiyama et al. (41) demonstrated that dietary supplementation with 3 × 10^5^ CFU/g probiotics exerted a significant effect on improving eggshell strength and thickness. Furthermore, a recent study revealed that dietary supplementation with 0.01% probiotics improved eggshell quality by regulating the expression of mineralization-related genes and the ion content in serum and eggshells (39). Collectively, these studies demonstrate improvements in eggshell quality. In contrast, Wang et al. (23) reported that supplementation with 0.05% compound probiotics significantly increased yolk weight but did not improve eggshell strength or thickness, highlighting that the result depends on probiotic formulation and dosage.

The improvement in eggshell strength can be attributed, on the one hand, to probiotics modulating the transport and absorption of trace minerals (39). These minerals play an essential role in the activity of calcium crystallization-related enzymes during eggshell formation (42). Additionally, relevant studies have confirmed that supplementing bioactive substances, such as probiotics and enzymes, leads to the redistribution of trace elements and an increased metabolic demand for major trace elements (43). On the other hand, the improvement in eggshell quality may result from increased intestinal calcium absorption (40). It has been reported that probiotics enhance calcium absorption and utilization significantly (44), and an increase in serum total calcium can promote eggshell formation and improve eggshell quality (39).

The intestine is the primary site for nutrient absorption, and intestinal health is crucial for the efficient absorption of nutrients and protection against harmful substances. Good intestinal morphological traits, including villus height, crypt depth, and the villus-to-crypt ratio, are important indicators of intestinal health (45). Numerous studies have confirmed that probiotic supplementation can improve intestinal health, promote the development of intestinal villi, and maintain favorable intestinal morphology (40, 46–49). In a recent study, Wang et al. (23) found that dietary supplementation with 0.05% multi-strain probiotics in laying hens significantly decreased jejunal crypt depth and increased the villus-to-crypt ratio. Additionally, Pan et al. (50) reported that supplementation with 0.02% probiotics in layer diets markedly increased jejunal villus height. Consistent with the aforementioned findings, our study found that dietary supplementation with a 0.1% probiotic-enzyme complex in laying hens significantly increased jejunal villus height and the villus-to-crypt ratio, indicating that the probiotic-enzyme complex supports intestinal development and health.

Notably, current research on probiotics or enzyme preparations supplementation in layer diets has primarily focused on laying performance, egg quality, and intestinal development (23, 27, 28, 30, 31), with few studies systematically elucidating host metabolic changes using multi-omics technologies. Therefore, we further analyzed the intestinal microbiota and serum metabolites of laying hens. The results revealed that differentially abundant metabolites across groups were significantly enriched in metabolic pathways, including biosynthesis of cofactors, biosynthesis of amino acids, and arginine biosynthesis. L-arginine and 4-hydroxybenzoic acid, as key metabolites in these pathways, showed higher relative abundance in the PEC group. Although arginine is considered a semi-essential or conditionally essential amino acid in many animal species (51), it is an essential amino acid for poultry (52–54). Arginine can be metabolized to produce nitric oxide (NO), exerting immunomodulatory and pathogen-inhibitory effects (55, 56). It also plays an important role in growth and development, energy metabolism, and protein synthesis (54, 57). Sun et al. (54) reported that arginine deficiency increases oxidative stress and intestinal damage, whereas arginine supplementation can alleviate the adverse effects of low-protein diets. A previous study has shown that dietary arginine supplementation is associated with improvements in laying performance and feed efficiency in laying hens. (58). Consistently, a recent study found that supplementing with 0.3% arginine significantly increased egg production rate and egg weight, and enhanced antioxidant and immune capacities (59). Arginine supplementation has been reported to increase eggshell thickness and improve eggshell quality (60), which is consistent with the findings of this study. In our study, enhanced arginine metabolism was observed in the PEC group, which may be related to the improvement in eggshell quality. However, some studies have not observed that arginine supplementation improves laying performance or eggshell quality (54, 61). This discrepancy may be attributed to differences in the supplementation level and the breed of laying hens. In addition, 4-hydroxybenzoic acid (a metabolite involved in the cofactor biosynthesis pathway) exhibits multiple biological activities, including glucose-lowering, anti-inflammatory, antiviral, and antioxidant effects (62), suggesting its potential role in promoting animal health. Therefore, in the present study, the improved laying performance may be associated with the elevated arginine levels and enhanced activity of the cofactor biosynthesis pathway.

The intestinal microbiota is essential for fermenting complex carbohydrates (e.g., host-indigestible non-starch polysaccharides and resistant starch), producing metabolites like short-chain fatty acids and vitamins that maintain intestinal health and regulate host metabolism (26, 33, 63, 64). Given this crucial role, dietary supplementation with prebiotics and probiotics has become a key strategy for enhancing intestinal health. These supplements can modulate the microbiota, improve host performance, and enhance colonization resistance against pathogens (39, 63, 65, 66). Consistent with prior evidence that probiotic supplementation increases beneficial bacteria in laying hens (33, 50, 65, 67), a similar shift was observed in our study. Specifically, genera such as *Lactobacillus*, *Bacillus*, *Faecalibacterium*, *Intestinimonas*, *unclassified Bacteroidia*, and *Bifidobacterium* dominated the microbiota in the PEC group. Notably, *g__Lactobacillus* was identified as a key discriminatory genus by both random forest and LEfSe analyses.

*Lactobacillus* is a key intestinal probiotic that regulates gut pH by producing lactic acid and inhibits pathogenic bacteria. The functional benefits of Lactobacillus are well established. Zhao et al. (68) isolated *Lactobacillus rhamnosus* GG from indigenous chicken breeds and demonstrated its capacity to enhance growth, reduce inflammation, and improve antioxidant capacity and intestinal health in laying hens. Similarly, Liu et al. (69) reported that *L. rhamnosus* GG increased eggshell strength and thickness, as well as overall production performance. Additionally, *Lactobacillus salivarius* SNK-6 has been shown to promote intestinal development, production performance, and both antioxidant and immune capacities (70). Notably, *g__Lactobacillus* is closely associated with L-arginine synthesis (71, 72). Thus, the enrichment of probiotics such as *g__Lactobacillus* observed in this study may have contributed to improved laying performance, potentially by enhancing L-arginine synthesis.

Although our findings are promising, several limitations due to experimental conditions should be acknowledged. The sample sizes for metabolomics and microbiomics were inconsistent; therefore, no correlation analysis was performed. The associations between arginine and the gut microbiota, as well as its effects on production performance, are inferred from relevant references. Targeted omics approaches may be required to further validate potential biomarkers. Additionally, we did not collect feces to determine nutrient digestibility; thus, the inference that the probiotic-enzyme complex improves laying performance by enhancing nutrient digestibility is supported only by literature evidence.

## 5 Conclusion

In summary, reducing dietary protein levels during the peak laying period causes a decline in laying performance and egg quality. Under the present experimental conditions, 0.1% PEC supplementation partially mitigated the adverse effects of a low-protein diet on laying performance and eggshell quality. These changes were associated with alterations in intestinal morphology, cecal microbiota, and serum metabolic profiles. Therefore, using the PEC may be an effective strategy to counteract the negative impacts of LP diets in laying hens.

## Data Availability

The original contributions presented in the study are included in the article/Supplementary material, further inquiries can be directed to the corresponding author.
