# Supplementary material for: Probiotic-enzyme complex improves laying performance in laying hens fed low-protein diets by modulating gut microbiota and host metabolism
Source: Front Vet Sci. 2026 May 29;13:1834201. doi: 10.3389/fvets.2026.1834201 (PMC13259988; doi:10.3389/fvets.2026.1834201)
Supplement: Supplementary file 1 [file Table_1.DOCX]

Supplementary Material

| **Table S1: Significantly differential metabolites between the PEC group and the LP group** | |
| --- | --- |
| **Index** | **Trend** |
| Arg-Gly | UP |
| (E)-4-[(1-Methoxy-1-Oxopropan-2-Yl) Amino]-4-Oxobut-2-Enoic Acid | UP |
| Oxoamide | UP |
| Citrusin Ii | UP |
| 2-Methylidene-3-Oxo-4A,7,8,8A-Tetrahydro-4H-1,4-Benzoxazine-7-Carboxylic Acid | UP |
| Carbonyloxycamptothecin | UP |
| 1,2,3,4-Tetrahydro-3-Isoquinolinecarboxylic Acid | UP |
| Methyl Isoquinoline-3-Carboxylate | UP |
| 3'-Hydroxy Puerarin | UP |
| Indole-3-Acetylglycine | UP |
| N-Acetylgalactosamine 4,6-Disulfate | UP |
| Ethylmorphine | UP |
| Asn-Asp | UP |
| L-4-Chlorotryptophan | UP |
| Lamivudine | UP |
| 1,3-Dimethylthieno[2,3-C] Pyrazole-5-Carbohydrazide | UP |
| Linsitinib | UP |
| 5-Amino-6-(5'-Phosphoribitylamino) Uracil | UP |
| 2,3,4,9-Tetrahydro-1H-Pyrido[3,4-B] Indol-1-One | UP |
| Lycoperodine I | UP |
| Gly-Trp | UP |
| (+/-)-Tryptophan | UP |
| Nicotyrine | UP |
| 3,6-Dihydroxy-8-Methoxy-1-Methyl-2,3-Dihydro-1H-Cyclopenta[C]Isochromen-5-One | UP |
| Riboflavin | UP |
| 3-Methyldioxyindole | UP |
| Cotinine Glucuronide | UP |
| Gly-Arg | UP |
| Trans-4-Carboxymethylenebut-2-En-4-Olide | UP |
| Alanylasparagine | UP |
| Niacinamide | UP |
| N2-Acetylornithine | UP |
| Isoxanthopterin | UP |
| 4,5-Dihydroorotic Acid | UP |
| Pyroglutamyl-Glutamyl-Proline Amide | UP |
| (8-Hydroxy-3-Methyl-1-Oxo-3,4-Dihydroisochromen-5-Yl) Hydrogen Sulfate | UP |
| 5-Ethyl-4-Methyloxazole | UP |
| Taxiphyllin | UP |
| 2-Methylindoline | UP |
| 4-Methylhippuric Acid | UP |
| Oxolinic Acid | UP |
| T0070907 | UP |
| Nalidixic Acid | UP |
| 5,6-Dihydro-5-Azacytidine | UP |
| 5-Phenyl-1,3-Oxazinane-2,4-Dione | UP |
| 4-Vinylphenol Sulfate | UP |
| Isatoribine | UP |
| Lithocholate 3-O-Glucuronide | UP |
| Cucurbitacin C | UP |
| Oleoyl Serotonin | UP |
| Dodecyl-Beta-D-Maltoside | UP |
| Metyrapone | UP |
| Arg-Arg-Lys | UP |
| Cholylglutamine | UP |
| L-Arginine | UP |
| Thymopentin | UP |
| Oxyfluorfen | UP |
| Amrinone | UP |
| Gly Ile | UP |
| 4-Hydroxybenzoic Acid | UP |
| 3-Methylindole | UP |
| Brincidofovir | UP |
| Ferulic Acid | UP |
| Ziprasidone | down |
| Ethyl Gallate | down |
| Ricinine | down |
| Neuromedin B (4-10) | down |
| N-Alpha-Acetyl-L-Lysine | down |
| 1-(2-Furylmethyl)-5-Oxopyrrolidine-3-Carboxylic Acid | down |
| 3-Oxovalproic Acid | down |
| Acetone Cyanohydrin | down |
| Delpazolid | down |
| 2-Methylhippuric Acid | down |
| Synephrine | down |
| (S)-Spinacine | down |
| Abscisic Acid | down |
| Alpha-Eleostearic Acid | down |
| Enalapril | down |
| S-Sulfanylglutathione | down |

| **Table S2: Significantly differential metabolites between the PEC group and the HP group** | |
| --- | --- |
| **Index** | **Trend** |
| 25-O-Desacetyl Rifabutin | up |
| Dibenzylamine | up |
| N-(2-Phenylethyl) Acetamide | up |
| N-Acetyltryptamine | up |
| Prostaglandin A1 | up |
| Bicyclo Prostaglandin E2 | up |
| R-Deprenyl N-Oxide | up |
| Asn-Asp | up |
| Oplopandiol | up |
| Desmethyl Nefopam | up |
| Prostaglandin D2 | up |
| 15-Deoxy-Delta-12,14-Prostaglandin J2 | up |
| N-Acetylhistamine | up |
| Glu-Thr-Ala | up |
| 11-Hpode | up |
| 6Beta-Prostaglandin I1 | up |
| 9-Oxo-11R,15S-Dihydroxy-1A,1B-Dihomo-13E-Prostaenoic Acid | up |
| (9Z)-4,12,12-Trimethyl-5-Oxatricyclo [9.1.0.04,6] Dodec-9-Ene-9-Carboxylic Acid | up |
| Dg (Pgd2/I-18:0/0:0) | up |
| 12-Hhtre | up |
| 2-(E-3-Heptneyl)-3, 6-Dihydroxy-5-(3-Methyl-2-Butenyl) Benzaldehyde | up |
| Cortolone | up |
| Lithocholate 3-O-Glucuronide | up |
| 5-(4'-Hydroxyphenyl)-Gamma-Valerolactone 4'-Sulfate | up |
| L-Arginine | up |
| Carbocyclic Thromboxane A2 | up |
| Emopamil | up |
| 8(S)-Hetre | up |
| 8-Iso-15-Keto-Prostaglandin-F2Beta | up |
| Lactupicrin | up |
| Thromboxane B2 | down |
| Alpha-Carboxy-Delta-Decalactone | down |
| 4-Hydroxybenzoic Acid | down |
| Beta-D-Glucosamine | down |
| Glu-Thr | down |
| 15-Hete | down |
| Alpha-Eleostearic Acid | down |
| Deoxycorticosterone Acetate | down |

| **Table S3: Significantly differential metabolites between the LP group and the HP group** | |
| --- | --- |
| **Index** | **Trend** |
| Formyllysine | down |
| 1,2,3,4-Tetrahydro-3-Isoquinolinecarboxylic Acid | down |
| Methyl Isoquinoline-3-Carboxylate | down |
| L-4-Chlorotryptophan | down |
| Lamivudine | down |
| 1,3-Dimethylthieno[2,3-C] Pyrazole-5-Carbohydrazide | down |
| Lycoperodine I | down |
| Gly-Trp | down |
| (+/-)-Tryptophan | down |
| Trans-4-Carboxymethylenebut-2-En-4-Olide | down |
| N2-Acetylornithine | down |
| 2-Methylindoline | down |
| 5-Phenyl-1,3-Oxazinane-2,4-Dione | down |
| Dodecyl-Beta-D-Maltoside | down |
| L-Arginine | down |
| Oxyfluorfen | down |
| Amrinone | down |
| 3-Methylindole | down |
| 2-Methylhippuric Acid | down |
| S-Sulfanylglutathione | down |

| **Table S4: Alpha diversity of cecal microorganisms** | | | | | |
| --- | --- | --- | --- | --- | --- |
| sample | shannon | simpson | chao1 | ace | goods_coverage |
| LP-1 | 4.853 | 0.971 | 1822.679 | 1895.238 | 0.998 |
| LP-2 | 4.667 | 0.970 | 1561.757 | 1629.533 | 0.998 |
| LP-3 | 4.587 | 0.964 | 1402.659 | 1454.396 | 0.998 |
| HP-1 | 4.883 | 0.977 | 1596.981 | 1655.199 | 0.998 |
| HP-2 | 5.075 | 0.983 | 1686.464 | 1755.404 | 0.998 |
| HP-3 | 4.614 | 0.966 | 1569.942 | 1655.510 | 0.998 |
| PEC-1 | 4.804 | 0.977 | 1454.186 | 1510.385 | 0.998 |
| PEC-2 | 4.596 | 0.975 | 1284.622 | 1310.186 | 0.998 |
| PEC-3 | 4.600 | 0.971 | 1470.190 | 1545.369 | 0.998 |


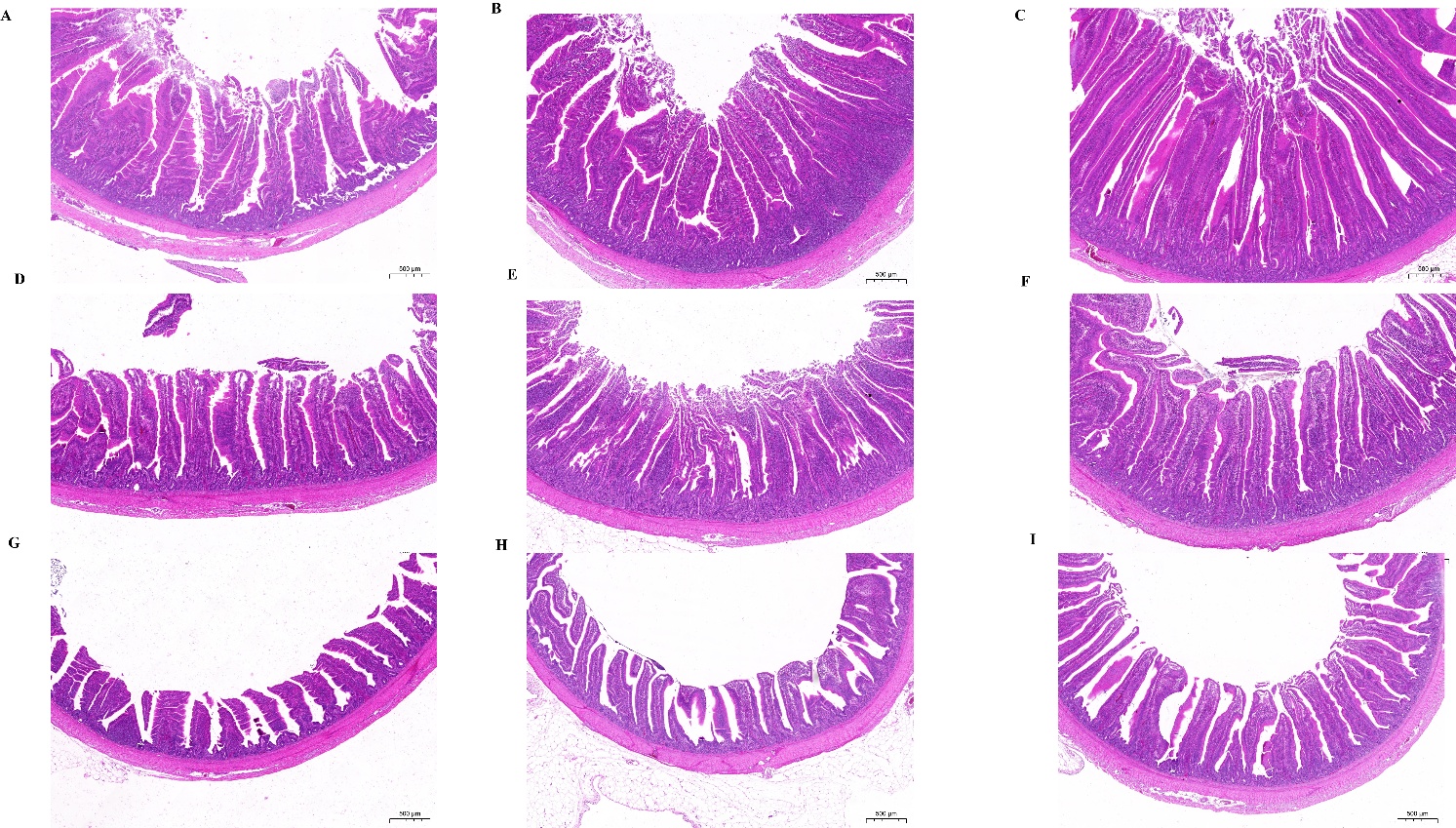


**Supplementary Figure 1: Intestinal development section images.** Panels A, B, and C show duodenal sections of the LP, HP, and PEC groups, respectively; panels D, E, and F show jejunal sections of the LP, HP, and PEC groups, respectively; panels G, H, and I show ileal sections of the LP, HP, and PEC groups, respectively.
